# Incidence of acute kidney injury and use of renal replacement therapy in intensive care unit patients in Indonesia

**DOI:** 10.1186/s12882-020-01849-y

**Published:** 2020-05-20

**Authors:** Jonny Jonny, Moch Hasyim, Vedora Angelia, Ayu Nursantisuryani Jahya, Lydia Permata Hilman, Venna Febrian Kusumaningrum, Nattachai Srisawat

**Affiliations:** 1 Division of Nephrology, Department of Internal Medicine, Gatot Soebroto Indonesia Central Army Hospital, Jakarta, Indonesia; 2 Department of Anesthesiology and Reanimation, Gatot Soebroto Indonesia Central Army Hospital, Jakarta, Indonesia; 3 Division of Nephrology, Department of Medicine, Faculty of Medicine, Chulalongkorn University, Bangkok, Thailand; 4 Critical Care Nephrology Research Unit, Faculty of Medicine, Chulalongkorn University, Bangkok, Thailand; 5 Academic of Science, Royal Society of Thailand, Bangkok, Thailand; 6 Tropical Medicine Cluster, Chulalongkorn University, Bangkok, Thailand; 7 Excellence Center for Critical Care Nephrology, King Chulalongkorn Memorial Hospital, Bangkok, Thailand; 8 Excellence Center for Critical Care Medicine, King Chulalongkorn Memorial Hospital, Bangkok, Thailand

**Keywords:** Acute kidney injury, Intensive care unit, Incidence, Survival, Renal replacement therapy

## Abstract

**Background:**

Currently, there is limited epidemiology data on acute kidney injury (AKI) in Indonesia. Therefore, we assessed the incidence of AKI and the utilization of renal replacement therapy (RRT) in Indonesia.

**Methods:**

Demographic and clinical data were collected from 952 ICU participants. The participants were categorized into AKI and non-AKI groups. The participants were further classified according to the 3 different stages of AKI as per the Kidney Disease Improving Global Outcome (KDIGO) criteria.

**Results:**

Overall incidence of AKI was 43%. The participants were divided into three groups based on the AKI stages: 18.5% had stage 1, 33% had stage 2, and 48.5% had stage 3. Primary diagnosis of renal disease and high APACHE II score were the risk factors associated with AKI (OR = 4.53, 95% CI: 1.67–12.33, *p* = 0.003 and OR = 1.14 per 1 unit increase, 95% CI: 1.09–1.20, *p* < 0.001, respectively). Chronic kidney disease was the risk factor for severe AKI. Sepsis was the leading cause of AKI. Among the AKI participants, 24.6% required RRT. The most common RRT modalities were intermittent hemodialysis (71.7%), followed by slow low-efficiency dialysis (22.8%), continuous renal replacement therapy (4.3%), and peritoneal dialysis (1.1%).

**Conclusions:**

This study showed that AKI was a common problem in the Indonesian ICU. We strongly believe that identification of the risk factors associated with AKI will help us develop a predictive score for AKI so we can prevent and improve AKI outcome in the future.

## Background

Acute kidney injury (AKI) is a common problem and often associated with high morbidity and mortality rates worldwide. The International Society of Nephrology (ISN) has launched the 0 by 25 campaign. The purpose of this campaign is to have zero deaths from preventable or untreated AKI in resource limited setting (i.e., Africa, Latin America and Asia) by 2025 [1]. Recent report, mostly the data from resource sufficient countries, showed the incidence of AKI in ICU varies between 6 to 70% [1, 2].

On average, 5% of ICU patients with severe AKI require renal replacement therapy (RRT) [3]. AKI in ICU patients presents with diverse forms of clinical outcomes such as prolonged ICU stay by 3.5 days, reduced health-related quality of life, require mechanical ventilation, and have detrimental clinical consequences such as acid-base disorders, hypervolemia, hyperkalemia, uremia, impaired immune system, and depression. These variables are highly associated with the current mortality rates of AKI patients ranging from 40 to 60% [4, 5]. These findings suggested that ICU patients are highly susceptible to AKI and most of the time portend a poor prognosis.

There is limited epidemiological data on AKI in Indonesia and thus conducted this study to fill the gap. In addition, we assessed the risk factors associated with AKI and the incidence of acute RRT-treated in ICU.

## Methods

### Participants and data collection

This study was a prospective observational study conducted in an ICU of the Gatot Soebroto Indonesia Central Army Hospital, a tertiary care hospital with 800 beds capacity, located in Jakarta, Indonesia. This hospital is a central army hospital which serves not only Indonesian army but also general population.

**Fig. 1:**
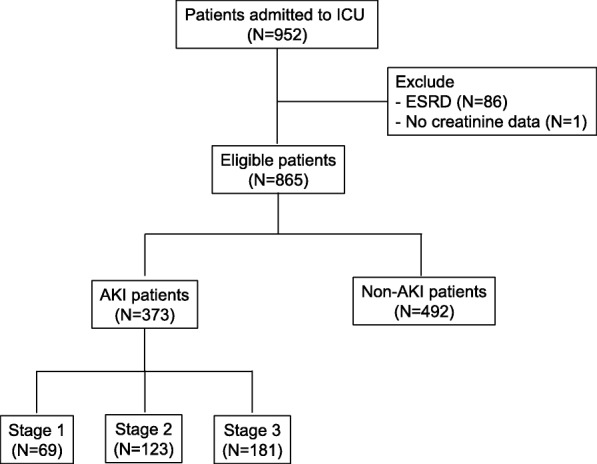
Study flow chart ICU, Intensive Care Unit; ESRD, End Stage Renal Disease; AKI, Acute Kidney Injury

All ICU patients > 15 years old were enrolled into the study from January 2017 until December 2018. Patients with end-stage renal disease (ESRD) on chronic dialysis were excluded (Fig. 1). For participants with multiple admissions, we only collected data from the first admission. The study protocol was reviewed and approved by Gatot Soebroto Indonesia Central Army Hospital ethics committee (IRB No. B/2212/VIII/2016). Informed consent was waived. This study is part of the Southeast Asia-Acute Kidney Injury (SEA-AKI) study that has been previously described by Srisawat et al. [6].

Demographic and clinical data from all of the participants were collected such as admission date, sex, age, body mass index (BMI), primary diagnosis at ICU admission, history of comorbid conditions, AKI stages, RRT modes, and the use of mechanical ventilation during ICU stay. The severity of participants was determined by the Acute Physiology and Chronic Health Evaluation II (APACHE II) and Sequential Organ Failure Assessment (SOFA) scoring system. Four RRT modes were used in this study: acute peritoneal dialysis (PD), intermittent hemodialysis (IHD), sustained low-efficiency dialysis (SLED), and continuous renal replacement therapy (CRRT). For each participant, we recorded how many times each RRT mode was used or as an initiation mode throughout January 2017–December 2018. The data were sequentially collected every day for the first 7 days of ICU admission and then collected weekly on days 14, 21, and 28.

### Definition and classification of acute kidney injury

The diagnosis of AKI was based on the KDIGO criteria [7]. For the baseline serum creatinine, we used the most recent available serum creatinine or serum creatinine within 1 year before hospital admission. If the participants had no available data for baseline serum creatinine, then we estimated the baseline serum creatinine using the lowest value between the serum creatinine at the time of hospital admission (admission serum creatinine) and the back calculation of serum creatinine from the Modification of Diet in Renal Disease (MDRD) equation using a glomerular filtration rate (GFR) of 75 mL/min/1.73m^2^ (MDRD serum creatinine) as recommended in the KDIGO guideline [8]. We also defined AKI stage 3 as severe AKI. We used International Classification of Diseases, 10th Revision coding to classify the primary diagnosis of ICU admission.

We defined renal recovery based on the Acute Dialysis Quality Initiative (ADQI) definition, that is, the participants are alive, had dialysis independent at hospital discharge and theirs serum creatinine on the last day of observation were less than 1.5 times compared to the baseline serum creatinine [9].

### Statistical analysis

Categorical data were presented as counts and percentages. Continuous data were presented as mean and standard deviation (SD) if normally distributed, or median with an interquartile range if non-normally distributed. Kaplan-Meier survival curves were also generated for each AKI class. The Chi-square test of independence was used to compare the proportions of different types of participants based on their AKI status. ANOVA was used to compare continuous data of the participants’ characteristics based on their AKI status. The univariate and multivariate logistic regression models were performed to examine the association between the factors and the outcomes. Factors, which had *p*-value < 0.20 from the univariate model and had no multicollinearity as measured by Variance Inflation Factor (VIF) statistic, were entered into the multivariate model. *P*-value < 0.05 was considered statistically significant. The statistical software used for all analyses was Stata version 14.0 (StataCorp LP., Texas).

## Results

Flow chart of the study was shown in Fig. 1. Among all study participants (*N* = 952), 87 were excluded because 1 participant did not have any creatinine data and 86 participants had ESRD. From 865 participants, almost half of them (43%) suffered from AKI. As for the severity of AKI, 48.5% of the participants had stage 3 AKI, 33% had stage 2 AKI and 18.5% had stage 1 AKI.

Among all AKI patients, the average age was 58 (± 15) years and 64.6% were male. The primary diagnosis upon ICU admission were most likely due to surgical-related disease (23.5%; *N* = 87), cardiovascular disease (19.2%; *N* = 71), and neurologic disease (15.9%; *N* = 59). The most common metabolic comorbidities among the AKI participants were hypertension (39.4%; *N* = 147) and diabetes mellitus (38.9%; *N* = 145). The severity scoring (APACHE II) and non-renal SOFA score were significantly higher in AKI participants (compared to non-AKI participants [17.1 vs 11.2, *p* < 0.001, and 5.0 vs 3.0, *p* < 0.001) (Table 1).

**Table 1:** Patients’ characteristics stratified by AKI status (*N* = 865)

Characteristics	Non-AKI (*N* = 492)	AKI (*N* = 373)	All (*N* = 865)	***P***-value
**Age, years, mean (SD)**	55 (15)	58 (15)	57 (15)	0.008
**Male sex, n (%)**	297 (60.4)	219 (58.7)	516 (59.7)	0.62
**Reimbursement**	(*N* = 486)	(*N* = 362)	(*N* = 848)	0.45^*^
- Government officer	261 (53.7)	190 (52.5)	451 (53.2)	
- Private health insurance	0 (0.0)	2 (0.6)	2 (0.2)	
- Social security system	211 (43.4)	159 (43.9)	370 (43.6)	
- Cash/self-pay	14 (2.9)	10 (2.8)	24 (2.8)	
- Unknown	0 (0.0)	1 (0.3)	1 (0.1)	
**BMI, n (%)**				< 0.001
- Underweight	14 (2.8)	16 (4.3)	30 (3.5)	
- Normal	421 (85.6)	287 (76.9)	708 (81.8)	
- Overweight	54 (11.0)	56 (15.0)	110 (12.7)	
- Obese	3 (0.6)	14 (3.8)	17 (2.0)	
**Primary diagnosis, n (%)**	(*N* = 491)	(*N* = 370)	(*N* = 861)	< 0.001
- Cardiovascular diseases	106 (21.6)	71 (19.2)	177 (20.6)	
- Endocrine Diseases	11 (2.2)	18 (4.9)	29 (3.4)	
- Gastrointestinal Diseases	13 (2.6)	18 (4.9)	31 (3.6)	
- Hematologic Diseases	1 (0.2)	1 (0.3)	2 (0.2)	
- Infectious Diseases	14 (2.9)	36 (9.7)	50 (5.8)	
- Neurologic Diseases	42 (8.6)	59 (15.9)	101 (11.7)	
- Oncologic Diseases	13 (2.6)	12 (3.2)	25 (2.9)	
- Renal Diseases	9 (1.8)	29 (7.8)	38 (4.4)	
- Respiratory Diseases	17 (3.5)	36 (9.7)	53 (6.2)	
- Rheumatologic Diseases	0 (0.0)	3 (0.8)	3 (0.3)	
- Surgical related diseases	265 (54.0)	87 (23.5)	352 (40.9)	
**Comorbidity, n (%)**				
- HT	210 (42.7)	147 (39.4)	357 (41.3)	0.33
- DM	114 (23.2)	145 (38.9)	259 (29.9)	< 0.001
- CKD	3 (0.6)	43 (11.5)	46 (5.3)	< 0.001*
- Cerebrovascular	4 (0.8)	11 (2.9)	15 (1.7)	0.017
- Malignancy	24 (4.9)	18 (4.8)	42 (4.9)	0.97
- CAD	52 (10.6)	19 (5.1)	71 (8.2)	0.004
**APACHE-II score, mean (SD)**	9.9 (4.4)	16.5 (7.3)	12.7 (6.7)	< 0.001
**Non-renal SOFA score, mean (SD)**	3.0 (1.6)	5.0 (2.5)	3.9 (2.3)	< 0.001
**Vasopressors, n (%)**	4 (0.8)	41 (11.0)	45 (5.2)	< 0.001
**Mechanical ventilation, n (%)**	85 (17.3)	185 (49.6)	270 (31.2)	< 0.001

*BMI* Body mass index, *DM* Diabetes mellitus, *CAD* Coronary artery disease, *CVD* Cerebrovascular disease, *APACHE II* Acute physiologic and chronic health evaluation II, *SOFA* Sequential organ failure assessment

^*^P-value from Fisher’s exact test

Risk factors associated with the development of AKI are shown in Table 2. Participants that were admitted to the ICU with renal disease as their primary diagnosis were 4.53 times (95% CI: 1.67–12.33, *p* = 0.003) more likely to develop AKI during ICU compared to those who did not have renal disease as their primary diagnosis. The odds of developing AKI in ICU was 1.14 times higher for every one unit increase in APACHEII score (OR = 1.14 per 1 unit increase, 95% CI: 1.09–1.20, *p* < 0.001).

**Table 2:** Risk factors for AKI development using logistic regression analysis. ^a^ (*N* = 850)

Characteristics^b^	Unadjusted OR (95% CI)	***P***-value	Adjusted OR (95% CI)	***P***-value
**Age, 10-year increment**	1.11 (1.02, 1.22)	0.019	0.90 (0.80, 1.02)	0.11
**Male sex**	0.93 (0.70, 1.22)	0.60		
**Reimbursement,*N*** **= 834**				
- Government officer	0.98 (0.74, 1.29)	0.87		
- Social security system	Reference			
- Private health insurance /Cash/self-pay	1.18 (0.53, 2.61)	0.69		
**BMI**				
- Underweight	1.60 (0.76, 3.36)	0.22	1.18 (0.48, 2.91)	0.73
- Normal	Reference		Reference	
- Overweight / obese	1.67 (1.14, 2.47)	0.009	1.55 (0.97, 2.49)	0.07
**Primary diagnosis,*N*** **= 846**				
- Cardiovascular diseases	0.90 (0.64, 1.26)	0.53		
- Endocrine Diseases	2.31 (1.08, 4.95)	0.032	0.97 (0.38, 2.49)	0.95
- Gastrointestinal Diseases	1.94 (0.94, 4.02)	0.07	1.20 (0.49, 2.99)	0.69
- Hematologic Diseases	1.37 (0.09, 21.99)	0.82		
- Infectious Diseases	3.81 (2.02, 7.17)	< 0.001	1.15 (0.51, 2.57)	0.74
- Neurologic Diseases	2.06 (1.35, 3.15)	0.001	0.97 (0.53, 1.77)	0.92
- Oncologic Diseases	1.27 (0.57, 2.83)	0.55		
- Renal Diseases	3.87 (1.61, 9.31)	0.003	4.53 (1.67, 12.33)	0.003
- Respiratory Diseases	2.92 (1.61, 5.32)	< 0.001	1.53 (0.72, 3.25)	0.27
- Surgical related diseases	0.27 (0.20, 0.37)	< 0.001	0.60 (0.39, 0.93)	0.021
**Comorbidity**				
- HT	0.88 (0.66, 1.16)	0.35		
- DM	2.05 (1.52, 2.77)	< 0.001	1.08 (0.73, 1.61)	0.71
- Cerebrovascular	3.83 (1.21, 12.13)	0.022	1.55 (0.39, 6.17)	0.53
- Malignancy	1.02 (0.55, 1.91)	0.95		
- CAD	0.47 (0.27, 0.81)	0.006	0.50 (0.26, 0.98)	0.042
**APACHE-II score**	1.21 (1.17, 1.25) per 1-point increase	< 0.001	1.14 (1.09, 1.20) per 1-point increase	< 0.001
**Non-renal SOFA score**	1.62 (1.49, 1.76) per 1-point increase	< 0.001	1.14 (1.00,1.31) per 1-point increase	0.050
**Vasopressors**	13.08 (4.61, 37.17)	< 0.001	2.99 (0.93, 9.62)	0.067
**Mechanical ventilation**	4.76 (3.49, 6.51)	< 0.001	1.29 (0.80, 2.08)	0.30

Chronic kidney disease (CKD) was not included in this analysis because there was small number of CKD patients in non-AKI group

*BMI* Body mass index, *DM* Diabetes mellitus, *CAD* Coronary artery disease, *CVD* Cerebrovascular disease, *APACHE II* Acute physiologic and chronic health evaluation I, *SOFA* Sequential organ failure assessment

^a^15 participants who had AKI as the primary diagnosis for ICU admission were excluded from the analysis

^b^All these parameters came from the first day of ICU admission

Table 3 showed the participants’ characteristics and risk factors for developing severe AKI. Out of 373 AKI participants, 48.5% had severe AKI. Based on the multivariate analysis using logistic regression, participants who developed severe AKI were 2.47 times more likely to be male, 9.47 times more likely to have CKD and 3.43 times more likely to have malignancy. They also were more likely to have higher APACHE II score (OR: 1.07; per 1-point increase, 95% CI: 1.01–1.13), compared to less severe AKI participants. Participants with severe AKI were also 5.41 times more likely to use vasopressors during their stay at the ICU. The goodness of fits by Hosmer-Lemeshow (H-L) test for model of AKI risk factors and model of AKI severity were H-L Chi2 = 3.64 (*p* = 0.89), and H-L Chi2 = 3.08 (*p* = 0.93), respectively.

**Table 3:** Patients’ characteristics and risk factors for severe AKI using logistic regression analysis (*N* = 373)

Characteristics^a^	Unadjusted OR (95% CI)	***P***-value	Adjusted OR (95% CI)	***P***-value
**Age, 10-year increment**	0.93 (0.81, 1.07)	0.284		
**Male sex**	1.93 (1.27, 2.94)	0.002	2.47 (1.48, 4.12)	0.001
**Reimbursement,*N*** **= 361**				
- Government officer	1.04 (0.68, 1.59)	0.85		
- Social security system	Reference			
- Private health insurance/Cash/self-pay	0.35 (0.09, 1.36)	0.13		
**BMI**				
- Underweight	2.32 (0.82, 6.56)	0.11	2.26 (0.7, 7.32)	0.18
- Normal	Reference		Reference	
- Overweight / obese	3.74 (2.10, 6.65)	< 0.001	2.26 (0.7, 7.32)	0.18
**Primary diagnosis,*N*** **= 370**				
- Cardiovascular diseases	0.78 (0.46, 1.31)	0.35		
- Endocrine Diseases	0.51 (0.19, 1.39)	0.19	0.41 (0.12, 1.44)	0.16
- Gastrointestinal Diseases	0.65 (0.25, 1.74)	0.40		
- Hematologic Diseases	NA			
- Infectious Diseases	2.00 (0.98, 4.07)	0.058	1.24 (0.51, 2.97)	0.64
- Neurologic Diseases	1.31 (0.75, 2.28)	0.35		
- Oncologic Diseases	1.06 (0.33, 3.34)	0.92		
- Renal Diseases	3.00 (1.30, 6.97)	0.010	1.5 (0.5, 4.56)	0.47
- Respiratory Diseases	1.36 (0.68, 2.71)	0.38		
- Rheumatologic Diseases	2.12 (0.19, 23.62)	0.54		
- Surgical related diseases	0.50 (0.30, 0.82)	0.006	0.8 (0.43, 1.49)	0.48
**Comorbidity**				
- HT	1.29 (0.85, 1.96)	0.23		
- DM	1.70 (1.11, 2.58)	0.014	1.15 (0.65, 2.02)	0.63
- CKD	12.91 (4.51, 36.96)	< 0.001	9.47 (3.1, 28.91)	< 0.001
- Cerebrovascular	0.39 (0.10, 1.48)	0.17		
- Malignancy	2.20 (0.81, 5.99)	0.12	3.43 (1.11, 10.57)	0.032
- CAD	0.95 (0.38, 2.40)	0.92		
**APACHE-II score**^a^	1.07 (1.04, 1.10) per 1-point increase	< 0.001	1.07 (1.01, 1.13) per 1-point increase	0.018
**Non-renal SOFA score**^a^	1.14 (1.05, 1.24) per 1-point increase	0.002	0.92 (0.78, 1.08) per 1-point increase	0.29
**Vasopressors**	9.29 (3.55, 24.26)	< 0.001	5.41 (1.8, 16.29)	0.003
**Mechanical ventilation**	1.70 (1.13, 2.55)	0.012	1.31 (0.69, 2.49)	0.41

NA: not included in the multivariable model even though the *P*-value was < 0.20 because there were limited data

*BMI* Body mass index, *DM* Diabetes mellitus, *CAD* Coronary artery disease, *CVD* Cerebrovascular disease, *APACHE II* Acute physiologic and chronic health evaluation II, *SOFA* Sequential organ failure assessment

^a^All of these parameters came from the first day of ICU admission

We also explored the etiology of AKI (Table 4). Sepsis was the leading cause of AKI (206, 55.2%). While, AKI secondary to obstetric complication occurred in only 3 cases (0.8%) (Table 4).

**Table 4:** Etiology of AKI

Etiology of AKI	N (%)
Renal hypoperfusion^a^	76 (20.4)
Liver^b^	1 (0.3)
Cardiovascular^c^	71 (19.0)
Obstructive uropathy	16 (4.3)
Pregnancy^d^	3 (0.8)
Sepsis	206 (55.2)

^a^Renal hypoperfusion included hypovolemic shock, dehydration, renal artery stenosis

^b^Liver included hepatorenal syndrome, liver cirrhosis, acute liver failure, acute hepatitis

^c^Cardiovascular included myocardial infarction, heart failure, heart valve disorder, pulmonary embolism, bacterial endocarditis, and cardiorenal syndrome

^d^Pregnancy included preeclampsia

Among those participants who received RRT support, IHD was the most common procedure used as the initial mode of RRT in ICU (66 procedures, 71.7%), followed by SLED (21 procedures, 22.8%), then CRRT (4 procedures, 4.3%) and acute PD (1 procedure, 1.1%) (Table 5).

**Table 5:** Modes of RRT (initiation mode if many were used simultaneously)

Mode of RRT	N (%)
IHD	66 (71.7)
CRRT	4 (4.34)
PD	1 (1.08)
SLED	21 (22.8)

*IHD* Intermittent hemodialysis, *CRRT* Continuous renal replacement therapy, *PD* Peritoneal dialysis, *SLED* Sustained low efficiency dialysis

**Fig. 2:**
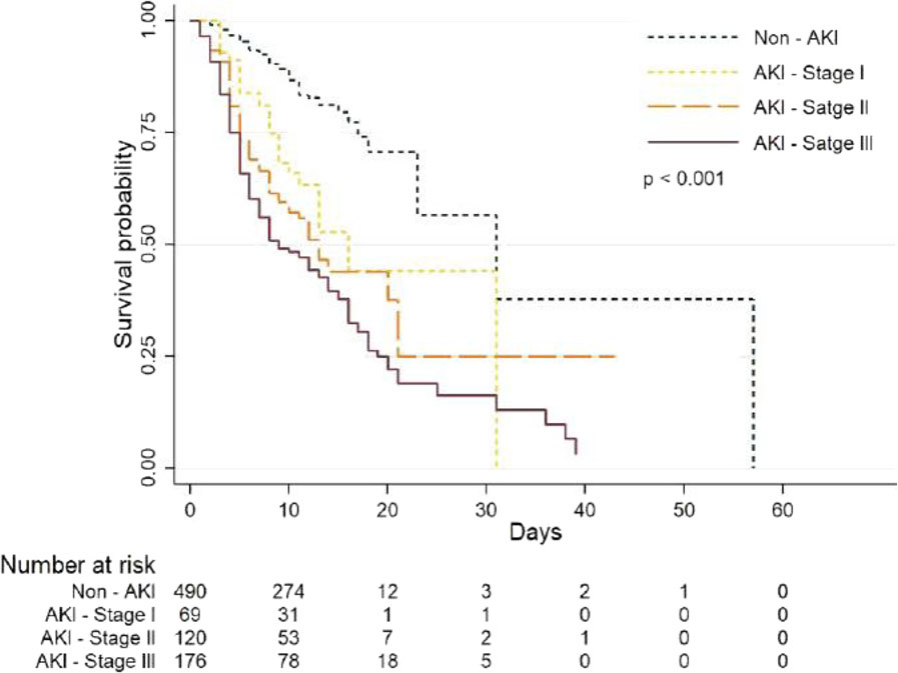
Hospital mortality based on the Kaplan-Meier survival curves for each AKI stage, *N* = 855 (excluded 10 events time missing)

Survival analysis was acquired by using the Kaplan-Meier analysis. It was tabulated for 60 days in the hospital and 28 days in the ICU. In Fig. 2, the curve estimated the overall survival based on the different stages of AKI while in the hospital. AKI stage 3 was associated with a significant decrease in cumulative survival probability. The median survival time for non-AKI, AKI stage 1, 2, and 3 were 31, 16, 13, and 9 days, respectively (*p* < 0.001).

The renal recovery rate at hospital discharge was only 19% (69 of 368 patients; 5 cases were excluded because the clinical outcome was unknown). For the non-recovery group (299 patients), the hospital mortality rate for AKI was 58% (215 of 368). There were 0.5% (2 of 368 patients) who were alive and dependent on dialysis. Eighty two out of 368 participants (22%) had persistent AKI.

## Discussion

Multiple studies have shown the detrimental impact of AKI on ICU patients. Unfortunately, the etiology and the mechanisms of AKI remain unclear. Various reasons such as infection, trauma and other problems that prompt the patient to be admitted into ICU could induce AKI or worsen any pre-existing condition of AKI [1]. Therefore, it is important to have AKI epidemiology data, including the risk factors that may cause AKI. This set of information will allow the country, especially for Indonesia which has limited resources, to provide appropriate health care budget and management for these kinds of patients,. AKI has been reported to occur in 20–50% of the patients in ICUs around the world [10]. In Indonesia, at the Central Army Hospital of Gatot Soebroto, almost half of the ICU patients (43%) had AKI. This indicated that AKI was a common problem in Indonesian’s ICU (Fig. 1).

One of the most striking results in our study was the very high incidence of severe AKI among our cohort. This was in sharp contrast to other studies carried out in high-resource countries. Likewise, our findings are similar to those reported from less developed countries. A recent study conducted by Srisawat, et al. reported that the incidence of AKI stage 3 among patients in ICU was 28.9%; 16.4% of the patients at ICU had AKI stage 2 and 7.5% had AKI stage 1 [6]. Aside from that, they also showed that when the patients were admitted to ICU, most of the AKI patients progressed to AKI stage 3. This can be explained that AKI was not recognized in time so was not treated appropriately by the time the patients were admitted to the ICU. Hence, the incidence of AKI patients in developing countries are higher than those in developed country. For example, a recent study conducted in Australia reported that 37.1% of their patients in ICU developed AKI; 18.1% had AKI stage 1, 10.1% had AKI stage 2 and 8.9% had AKI stage 3 [11].

In our center, based on the multivariate analysis using logistic regression, the significant factors that were associated with severe AKI were male, CKD, malignancy, high APACHE II score, and use of a vasopressor (Table 3). Even though age was not significantly associated with severe AKI, however, we noticed that the older the participant was, the more severe the AKI was. For our study, the mean age of our participants with AKI was 58 years (± 15 years old). Our findings corroborated the results from a study conducted in Malaysia which showed that the mean age of the AKI patients was 53 years (± 16 years old) and that 56% of the patients had AKI stage 3, 25% had AKI stage 2 and 18% had AKI stage [12]. CKD was associated with severe AKI (9.47 times) (Table 3). In recent years, studies from different regions reported that CKD is a strong risk factor for the development of AKI, mainly in septic patients. Currently, CKD is found in 30% of the patients who develop AKI in ICU [13]. It is noteworthy that CKD had the highest percentage of association with AKI and was a strong predictor of developing AKI in critically ill patients.

By using the standard KDIGO criteria, which employs both the creatinine level and urine output data, we were able to study AKI epidemiological data both AKI incidence and risk factors of AKI in Indonesia. The finding from this study can be used as a reference data for other AKI studies conducted in Indonesia in the future. However, our study had some limitations. We collected data from only one site, at the Gatot Soebroto Hospital. Therefore, our data may not truly represent the incidence of AKI in all hospitalized ICU patients across Indonesia. Second, we only collected the data from ICU. Hence, it is possible that our participants could have developed AKI before ICU admission. Third, we only had baseline creatinine level from 9.9% of the participants. However, we were able to determine the baseline creatinine by choosing the reference creatinine from the lower value between the first serum creatinine on the day of admission and the creatinine from the MDRD formula which assumed that the GFR was at least 75 mL/min/1.73m^2^. We demonstrated that it was feasible to exclusively use the MDRD-derived values to generate the baseline serum creatinine levels for our participants [14–16]. We also know that the limitation of MDRD back calculation is based on the assumption that the participant did not have CKD. This was not a problem for our study. For us, our database has the CKD status of the participant before ICU admission. If the participant had a history of CKD, then we used the first available serum creatinine as the baseline serum creatinine level, not the MDRD back calculation. Fourth, we did not collect data on the drugs used by the participants which could affect the level of the serum creatinine. Last, we acknowledged that the timing of AKI onset in our study is unclear as only data in ICU is available in our database and, therefore, some AKI might occur before ICU admission. However, we preferred to use the term “incidence” rather than “prevalence” with the understanding of this limitation.

## Conclusion

In Indonesia, like other developing countries, nearly half of ICU patients were at risk of developing AKI. Of these AKI patients, 48.5% had severe AKI (AKI stage 3). Underlying disease of CKD is the strongest risk factors for severe AKI. Sepsis was the leading cause of AKI. With these results, we have identified AKI burden and risk factor of AKI in Indonesia. The strategies to improve national healthy policy should be encouraged for sustainable improvement in AKI care to achieve the goal of 0by25.

## Data Availability

The datasets used for the current study are available from the corresponding author if the requests are reasonable.

## Abbreviations

AKI: Acute kidney injury
RRT: Renal replacement therapy
KDIGO: Kidney Disease Improving Global Outcome
APACHE: Acute Physiology and Chronic Health Evaluation
ICU: Intensive care unit
ESRD: End Stage Renal Disease
SOFA: Sequential Organ Failure Assessment
PD: Peritoneal dialysis
IHD: Intermittent hemodialysis
SLED: Sustained low-efficiency dialysis
CRRT: Continuous renal replacement therapy
GFR: Glomerular filtration rate
MDRD: Modification of Diet in Renal Disease
ADQI: Acute Dialysis Quality Initiative
SD: Standard deviation
VIF: Variance Inflation Factor
CKD: Chronic kidney disease

## References

[1] Mehta RL, Cerda J, Burdmann EA, Tonelli M, Garcia-Garcia G, Jha V (2015). International Society of Nephrology’s 0by25 initiative for acute kidney injury (zero preventable deaths by 2025): a human rights case for nephrology. Lancet.

[2] Uchino S, Bellomo R, Morimatsu H, Morgera S, Schetz M, Tan I (2007). Continuous renal replacement therapy: a worldwide practice survey. Intensive Care Med.

[3] Uchino S, Kellum JA, Bellomo R, Doig GS, Morimatsu H, Morgera S (2005). Acute renal failure in critically ill patients: a multinational, multicenter study. JAMA.

[4] Dennen P, Douglas IS, Anderson R (2010). Acute kidney injury in the intensive care unit: an update and primer for the intensivist. Crit Care Med.

[5] Thakar CV, Christianson A, Freyberg R, Almenoff P, Render ML (2009). Incidence and outcomes of acute kidney injury in intensive care units: a Veterans Administration study. Crit Care Med.

[6] Srisawat N, Kulvichit W, Mahamitra N, Hurst C, Praditpornsilpa K, Lumlertgul N, et al. The epidemiology and characteristics of acute kidney injury in the Southeast Asia intensive care unit: a prospective multicentre study. Nephrol Dial Transplant. 2019. 10.1093/ndt/gfz087. [Epub ahead of print]. 10.1093/ndt/gfz087 31075172

[7] KDIGO A. Work Group (2012). KDIGO clinical practice guideline for acute kidney injury. Kidney Int Suppl.

[8] Srisawat N, Sileanu FE, Murugan R, Bellomo R, Calzavacca P, Cartin-Ceba R (2015). Variation in risk and mortality of acute kidney injury in critically ill patients: a multicenter study. Am J Nephrol.

[9] Chawla LS, Bellomo R, Bihorac A, Goldstein SL, Siew ED, Bagshaw SM (2017). Acute kidney disease and renal recovery: consensus report of the Acute Disease Quality Initiative (ADQI) 16 Workgroup. Nat Rev Nephrol.

[10] Srisawat N, Kellum JA (2011). Acute kidney injury: definition, epidemiology, and outcome. Curr Opin Crit Care.

[11] Bagshaw SM, George C, Bellomo R, Committe ADM (2008). A comparison of the RIFLE and AKIN criteria for acute kidney injury in critically ill patients. Nephrol Dial Transplant.

[12] Ralib AM, Nor MBM (2015). Acute kidney injury in a Malaysian intensive care unit: assessment of incidence, risk factors, and outcome. J Crit Care.

[13] Tejera D, Varela F, Acosta D, Figueroa S, Benencio S, Verdaguer C (2017). Epidemiology of acute kidney injury and chronic kidney disease in the intensive care unit. Rev Bras Ter Intensiva.

[14] Hoste EA, Clermont G, Kersten A, Venkataraman R, Angus DC, De Bacquer D (2006). RIFLE criteria for acute kidney injury are associated with hospital mortality in cr6tically ill patients: a cohort analysis. Crit Care.

[15] Wild S, Roglic G, Green A, Sicree R, King H (2004). Global prevalence of diabetes: estimates for the year 2000 and projections for 2030. Diabetes Care.

[16] Závada J, Hoste E, Cartin-Ceba R, Calzavacca P, Gajic O, Clermont G (2010). A comparison of three methods to estimate baseline creatinine for RIFLE classification. Nephrol Dial Transplant.

